# Photovoltaic driven thermo-electro dual module sustainable decontamination in soil

**DOI:** 10.1016/j.eehl.2025.100173

**Published:** 2025-07-23

**Authors:** Yongping Shan, Mingxiu Zhan, Bin Liu, Feng Liu, Wentao Jiao, Yongguang Yin

**Affiliations:** a Research Center for Eco-Environmental Sciences, Chinese Academy of Sciences, Beijing 100085, China; b College of Metrology and Measurement Engineering, China Jiliang University, Hangzhou 310018, China

**This study develops a Photovoltaic Thermo-Electro Dual Module System (PTEDMS) integrating electrical resistance heating, electrokinetic transport, and solar-thermal storage for soil decontamination. Machine learning strategies optimize photovoltaic energy allocation to overcome intermittency, enhance mass transport, and promote sustainable, low-carbon soil remediation.**

Organic contamination in soils has become a pressing environmental concern, with profound consequences for eco-environmental health [1,2]. Heat-based desorption approaches have shown promise in the rapid removal of organic compounds, while efforts have focused on reducing energy consumption and related carbon emissions [3]. Recent advances in heat transport mechanisms have refined energy utilization. Developments in thermal enhanced electric field-driven fluid flow and particle migration (i.e., electrokinetic) have overcome the limits of mass transport in micro- and nanopores [4]. Coupled with oxidation and biodegradation, these innovations have significantly reduced energy consumption. Although these advances have achieved both a reduction in energy consumption and better mass transport, the energy consumption can still reach 1500 ​MJ per ton [5,6]. Meanwhile, rapid progress of photovoltaic (PV) technologies is offering an option to minimize the carbon footprint of remediation [7]. By integrating photovoltaic with precise transport and degradation models, there is great potential to achieve soil remediation while contributing to low-carbon emission goals for ecosystem sustainability.

Optimizing thermal desorption involves refining inter-media heat transfer to accelerate pollutant volatilization. Electrical Resistance Heating (ERH) is an innovative electrothermal technique that utilizes subsurface electrodes to generate heat via the Joule heating of interstitial water. Combining linear voltage control, alternative/direct current switching, and variable-frequency output, it enables precise heat flux density regulation and a 30% reduction in carbon emission [8]. ERH serves as a foundation for hybrid approaches, e.g., electric field-driven particle transport (i.e., electrokinetic), chemical oxidation, thermophilic microbial metabolic degradation, etc. [9]. Electrokinetic enhances the transport of both contaminant and bio-degrader through the confined soil structure, which enhances the incidence of bio-engagement [10]. Hybridizing these strategies improves the overall remediation efficiency by 46%, while reducing energy consumption by 20% [11]. This enables the matching of the capability of renewable energy, such as PV, to ERH and electrokinetic transport, tailoring precise low-carbon soil remediation.

The advances in thermal and electrical process control align seamlessly with photovoltaic cogeneration, which provides a sustainable strategy for soil decontamination. Coupling with solar-thermal conversion, PV-driven electrokinetic transport, and biochemical modules, the PV-thermal hybrid system has great potential to minimize carbon emissions in soil remediation. However, it is a challenge to integrate and precisely manipulate the processes to allocate PV based on established models. Recent research on the environment-compatible multilevel ensemble learning (MEL) enables the integration of multiple machine learning models to deal with such multidimensional datasets [12]. Integration of MEL and sensor network-enabled real-time fidelity digital twins virtual replica has great potential to optimize the decontamination efficiency.

## 1 Advances in the optimization of ERH and hybrid processes

**Fig. 1:**
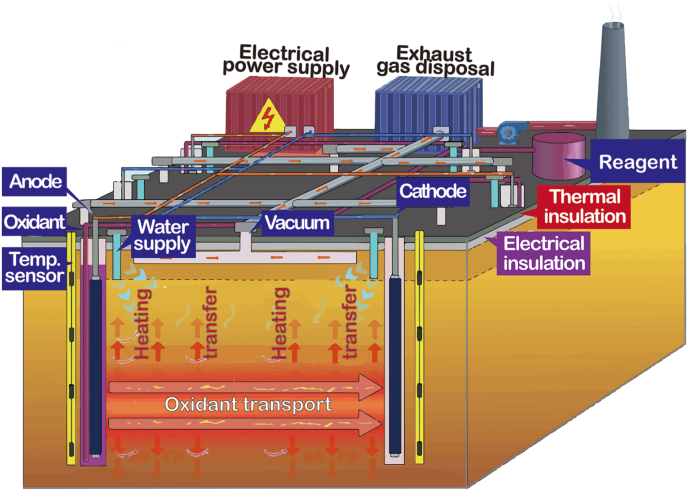
Electrical resistance heating and hybrid systems.

ERH and hybrid remediation processes that integrate multiple technologies have made notable strides in the treatment of soil and groundwater pollution (Fig. 1). By installing electrodes in interstitial water conductive contaminated areas, ERH elevates soil temperature to levels at which organic pollutants volatilize or degrade, thereby achieving contaminant removal. Compared with conventional methods, ERH demonstrates distinct advantages in heating coverage, precise temperature control, and adaptability to varying hydrogeological conditions, thus offering substantial potential for improving remediation efficiency.

The standalone application of ERH achieved excellent removal efficiency, while still encountering challenges due to high energy demands in confined areas [13]. Integrating ERH with in-situ chemical oxidation (ISCO) may enhance thermal activation of oxidants (e.g., persulfate), enhancing activation and utilization of oxidant by up to 49.3% [14]. Coupling ERH with electrokinetic has increased the transport of contaminant by 65% and degrading bacteria by 275% in micro-nano confinement pores [4,15]. This approach raised contaminant bioavailability in the most challenging confined areas to comply with remediation standards [16]. Heating together with ISCO and thermophilic microbes stimulated a 2.5-fold increase in degrading bacterial abundance and significantly enhanced contaminant removal [17,18]. Such advances have enabled optimizing the sequence of technologies to keep efficiency and reduce carbon emission, and established the groundwork for aligning with renewable energy such as PV.

## 2 Photovoltaic thermo-electro dual module sustainable soil remediation

**Fig. 2:**
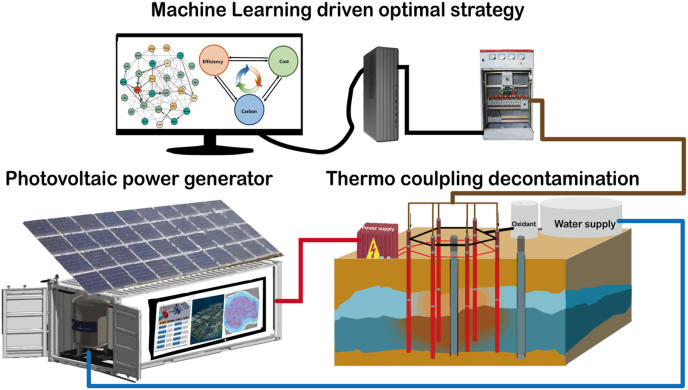
Schematic of Photovoltaic Thermo-Electro Dual Module System (PTEDMS).

Building on frontier research on thermal hybrid remediation and PV, we present an integrated photovoltaic thermo-electro dual module system (PTEDMS) for sustainable soil remediation (Fig. 2). Research found that 32% of PV power generation remains underutilized due to inadequate long-duration storage systems [19,20]. Hence, in the PV dual module remediation system, chemical batteries are replaced with water-based storage, achieving an energy exchange efficiency exceeding 85%. By optimizing the coordination between the heat storage of hot water and electro-processes in the PV-thermal hybrid solar system, PTEDMS allows uninterrupted operation driven by solar energy. Coupled with dynamic hot water cycling by pumps, this strategy sustains the power supply under cloudy conditions.

PTEDMS proposes an innovative solution for advancing soil pollution control and achieving low carbonization. Building on prior research in thermal and hybrid models, the system integrates thermo-electric energy supply, with hot water energy storage on the ground and pump-driven underground pipeline heating-exchange. This integrated approach enables efficient removal of organic contaminants while completely eliminating the carbon footprint of heating. Employing machine learning to optimize energy allocation between thermal-electrical conservation, the innovative system resolves the challenge of intermittent photovoltaic output and establishes a zero-carbon paradigm. This ensures a sustained power supply, provides a replicable pathway, and supports sustainable soil remediation.

## 3 Challenges and outlook

PTEDMS holds immense promise for sustainable soil and groundwater decontamination; however, this approach faces interdisciplinary and cross-scale challenges. A critical knowledge gap involves understanding the effects of diurnal photovoltaic power fluctuations on multiple removal pathways to achieve spatiotemporally precise remediation. Addressing this gap requires coordinated deployment of physico-chemical and biological modules across different scales, encompassing (i) the integration of macroscopic heat and mass transfer partial differential equations with molecular-scale desorption–oxidation kinetics, and (ii) the development of advanced, real-time methods to capture and respond to rapid shifts in power inputs and reaction conditions.

Machine learning is promising in integrating interdisciplinary models into neural networks based on mechanisms, including Arrhenius oxidation kinetics, Monod microbial thresholds, etc. Transfer learning will adapt these models to heterogeneous sites, enabling real-time photovoltaic energy allocation and fluid pumping. Multidimensional heuristic graph structure further visualizes and quantifies the interactions in the “black box” of machine learning. Together with these approaches, the PV-thermal hybrid system may provide sustainable, precise, low-carbon soil remediation.

## CRediT authorship contribution statement

**Yongping Shan:** Writing – original draft, Visualization, Validation, Software, Project administration, Methodology, Investigation, Funding acquisition, Data curation, Conceptualization. **Mingxiu Zhan:** Writing – review & editing, Validation, Methodology, Formal analysis, Conceptualization. **Bin Liu:** Writing – review & editing, Validation, Software, Methodology, Conceptualization. **Feng Liu:** Writing – review & editing, Writing – original draft, Methodology, Investigation, Formal analysis. **Wentao Jiao:** Writing – review & editing, Supervision, Project administration, Methodology, Investigation, Funding acquisition, Formal analysis, Conceptualization. **Yongguang Yin:** Writing – review & editing, Visualization, Validation, Supervision, Conceptualization.

## Declaration of competing interests

The authors declare the following financial interests/personal relationships which may be considered as potential competing interests: Wentao Jiao reports financial support was provided by the Chinese Academy of Sciences. Wentao Jiao and Yongping Shan report financial support was provided by the National Natural Science Foundation of China. If there are other authors, they declare that they have no known competing financial interests or personal relationships that could have appeared to influence the work reported in this paper.
